# Genetic heterogeneity of motor neuropathies

**DOI:** 10.1212/WNL.0000000000003772

**Published:** 2017-03-28

**Authors:** Boglarka Bansagi, Helen Griffin, Roger G. Whittaker, Thalia Antoniadi, Teresinha Evangelista, James Miller, Mark Greenslade, Natalie Forester, Jennifer Duff, Anna Bradshaw, Stephanie Kleinle, Veronika Boczonadi, Hannah Steele, Venkateswaran Ramesh, Edit Franko, Angela Pyle, Hanns Lochmüller, Patrick F. Chinnery, Rita Horvath

**Affiliations:** From the MRC Centre for Neuromuscular Diseases and John Walton Muscular Dystrophy Research Centre, Institute of Genetic Medicine (B.B., H.G., T.E., J.D., A.B., V.B., H.S., E.F., A.P., H.L., P.F.C., R.H.), and Institute of Neuroscience (R.G.W., J.M.), Newcastle University, Newcastle upon Tyne; Bristol Genetics Laboratory (T.A., M.G., N.F.), Pathology Sciences, North Bristol NHS Trust, Southmead Hospital; Medical Genetic Center (S.K.), Munich, Germany; Department of Paediatric Neurology (V.R.), Royal Victoria Infirmary, Newcastle upon Tyne Foundation Hospitals NHS Trust; Nuffield Department of Clinical Neurosciences (E.F.), University of Oxford; and Department of Clinical Neurosciences (P.F.C.), Cambridge Biomedical Campus, University of Cambridge, UK.

## Abstract

**Objective:**

To study the prevalence, molecular cause, and clinical presentation of hereditary motor neuropathies in a large cohort of patients from the North of England.

**Methods:**

Detailed neurologic and electrophysiologic assessments and next-generation panel testing or whole exome sequencing were performed in 105 patients with clinical symptoms of distal hereditary motor neuropathy (dHMN, 64 patients), axonal motor neuropathy (motor Charcot-Marie-Tooth disease [CMT2], 16 patients), or complex neurologic disease predominantly affecting the motor nerves (hereditary motor neuropathy plus, 25 patients).

**Results:**

The prevalence of dHMN is 2.14 affected individuals per 100,000 inhabitants (95% confidence interval 1.62–2.66) in the North of England. Causative mutations were identified in 26 out of 73 index patients (35.6%). The diagnostic rate in the dHMN subgroup was 32.5%, which is higher than previously reported (20%). We detected a significant defect of neuromuscular transmission in 7 cases and identified potentially causative mutations in 4 patients with multifocal demyelinating motor neuropathy.

**Conclusions:**

Many of the genes were shared between dHMN and motor CMT2, indicating identical disease mechanisms; therefore, we suggest changing the classification and including dHMN also as a subcategory of Charcot-Marie-Tooth disease. Abnormal neuromuscular transmission in some genetic forms provides a treatable target to develop therapies.

Distal hereditary motor neuropathies (dHMN) are clinically and genetically heterogeneous disorders caused by lower motor neuron dysfunction.^1^ The classic phenotype of dHMN is a length-dependent motor weakness and atrophy, initially affecting the intrinsic foot muscles and the peroneal compartment of the leg, frequently leading to foot deformities such as pes cavus, pes planus, and clawing of the toes.^2^ The majority of the cases show a slowly progressive disease course gradually involving proximal leg muscles or affecting intrinsic hand muscles. Variable age at onset, diverse clinical course, and associated neurologic features complicate the phenotype and aid the clinical subclassification. Congenital or infantile-onset forms of weakness and atrophy of the feet and legs with no or very slow disease progression were grouped as spinal muscular atrophy with lower extremity dominance (SMA-LED). Other clinical subgroups of dHMN were classified on the basis of additional features such as diaphragmatic paralysis (dHMN-IV), upper limb predominance (dHMN-V), SMA with respiratory distress (SMARD1 or dHMN-VI), adult-onset with vocal cord palsy (dHMN-VII), and pyramidal features or X-linked inheritance.^3^

Gene identification in dHMN was previously based on linkage studies in rare extended families. However, recent development of next-generation technology enabled studying isolated patients or small families, and a large number of new disease genes and novel phenotypes have been discovered.^4^ Compared to the 7 genes and 13 chromosomal loci 10 years ago,^5,6^ to date we acknowledge around 30 genes responsible for autosomal dominant, recessive, and X-linked forms of dHMN. To identify the molecular cause of motor neuropathies, we performed detailed clinical investigations and state of the art genetic studies in a large, population-based cohort from the North of England.

## METHODS

### Patients.

Between 2010 and 2015, we selected 105 patients out of 461 diagnosed with inherited neuropathies in our genetic neuropathy clinic at Newcastle Hospitals NHS Foundation, which is the catchment area of a total population of 2.99 million. Inclusion of patients was based on the presence of a motor neuropathy/neuronopathy with no or only subclinical sensory changes on electrophysiology. Acquired causes were excluded by detailed laboratory analysis/no response on immunosuppressive therapy. Some affected family members of patients with a confirmed genetic diagnosis were included in this cohort, even if no electrophysiologic data were available.

### Standard protocol approvals, registrations, and patient consents.

Participants provided written informed consent, approved by local research ethics committees, for all experiments using human participants and for photographs that may be published.

### Electrophysiology.

We performed electrophysiologic examination in 90 patients according to standard techniques and interpreted by the same expert neurophysiologists. Eight family members did not undergo electrophysiologic studies; 7 patients were tested at different hospitals. Motor and sensory nerve conduction studies (NCS) were performed and qualitative and quantitative analysis of motor unit potentials and spontaneous activity were assessed on EMG. Patient classification into study groups was based on these analyses. Additional assessment of repetitive nerve stimulation and single-fiber EMG were carried out in 27 patients, and the percentage increment or decrement was calculated. EMG was performed using Natus Neurology (Pleasanton, CA) disposable 30-G concentric needles with a bandpass range of 10–10,000 Hz. For single-fiber EMG studies, the low pass filter was increased to 2,000 Hz and the percentage of fiber pairs showing increased jitter or blocking was calculated.

### Molecular genetic analysis.

DNA was obtained from peripheral blood. Initially, *PMP22* deletion/duplication, *MFN2*, and *GJB1* were excluded in all patients. The multigene panel assay utilizing next-generation sequencing (NGS) was carried out in 46 index patients in the Bristol Genetics Laboratory following the UK Genetic Testing Network approved criteria. Variant classification was based on Association for Clinical Genetic Science Practice Guidelines (2013). We performed whole exome sequencing (WES) in 40 patients. DNA was fragmented, exome-enriched, and sequenced (Illumina [San Diego, CA] TruSeq 62 Mb and HiSeq 2000, 100 bp paired-end reads). Bioinformatic analysis included duplicate sequence read removal with FastUniq,^7^ alignment to UCSC hg19 with BWA^8^ variant detection with FreeBayes, and variant annotation with ANNOVAR. Variants were annotated as exonic/splicing, excluding synonymous variants, with a rare minor allele frequency (MAF < 0.01) in the context of genotype (heterozygous MAF < 0.001; homozygous MAF < 0.01) in several databases downloaded via ANNOVAR (ExAC, NHLBI_ESP6500, cg69) and also in 281 in-house exomes. Protein prediction and evolutionary sequence conservation algorithms downloaded via ANNOVAR were used to analyze the in silico effects following the guidelines of the American College of Medical Genetics and Genomics.^9^ Variants were defined as confirmed pathogenic if they were previously shown to be pathogenic or if they were novel variants in a known neuropathy-associated gene, predicted to be deleterious (affect protein structure or function, highly conserved), and segregated with the disease in at least 1 additional affected family member.^10^ Highly conserved, in silico deleterious rare sequence variants of known or novel genes were determined as possible pathogenic if segregation studies involving at least one additional affected family member could not be carried out. Putative pathogenic variants were confirmed by Sanger sequencing and were tested for segregation.

## RESULTS

### Clinical presentation.

We identified 105 patients from 73 families with inherited motor neuropathies. Based on neurologic and electrophysiologic findings, all patients were classified into 1 of 3 subgroups: dHMN, motor predominant Charcot-Marie-Tooth disease (CMT2) (motor CMT2), and motor neuropathy with additional neurologic symptoms (hereditary motor neuropathy [HMN] plus). Diagnosis of dHMN was based on preserved sensory nerve studies with normal or reduced compound motor unit action potentials or neurogenic changes on EMG examination.^2^ Patients with dHMN but with decreased sensory nerve action potentials, indicative of an accompanying sensory axonopathy, were grouped as motor CMT2. We considered HMN plus when motor neuropathy was the leading feature, but was accompanied by other neurologic symptoms. NCS data of all patients are available on request.

All patients seen in our center were from our catchment area (2.99 million people in 2011 UK census), leading to a minimum prevalence of dHMN as 2.14 affected individuals per 100,000 inhabitants (95% confidence interval [CI] 1.62–2.66).

**Figure 1:**
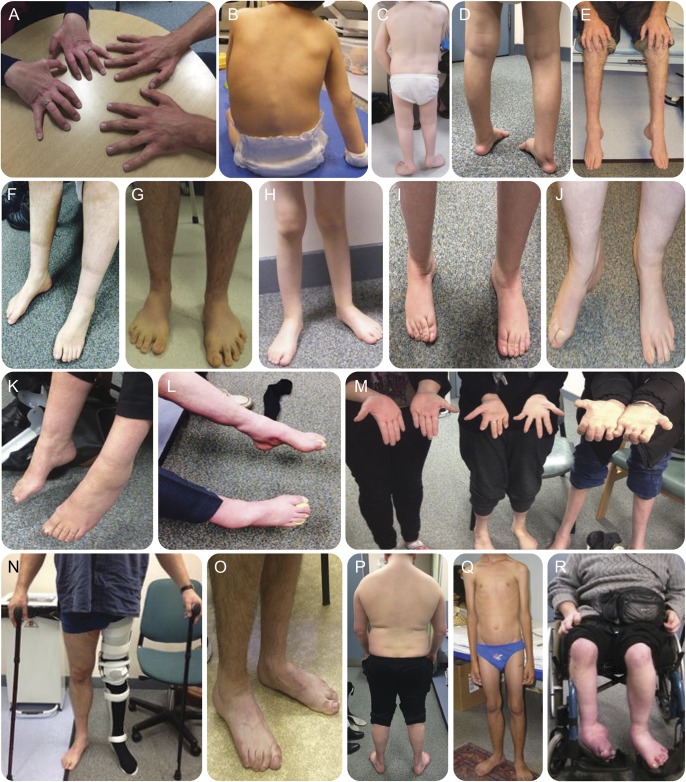
Clinical heterogeneity of different forms of hereditary motor neuropathy (HMN) Distal hereditary motor neuropathy (dHMN) (top 2 rows), motor Charcot-Marie-Tooth disease 2 (CMT2) (third row), and HMN plus (bottom row): (A) patients 2 and 3: *GARS*; (B) patient 19: *IGHMBP2*; (C) patient 23: *TRPV4*; (D) patient 32: *IGHMBP2*; (E) not yet diagnosed dHMN; (F) patient 18: *DYNC1H1*; (G) patient 10: *SYT2*; (H) patient 16: *BICD2*; (I, J) not yet diagnosed spinal muscular atrophy lower extremity dominance; (K) patient 42: *AARS*; (L) patient 39: *AARS*; (M) family 22: *DNM2*; (N) patient 51: *FUS*; (O) patient 53: *ATP7A*; (P) patient 57: *TBX5*; (Q) patient 58: *STAT5B*; (R) not yet diagnosed HMN plus.

**Figure 2:**
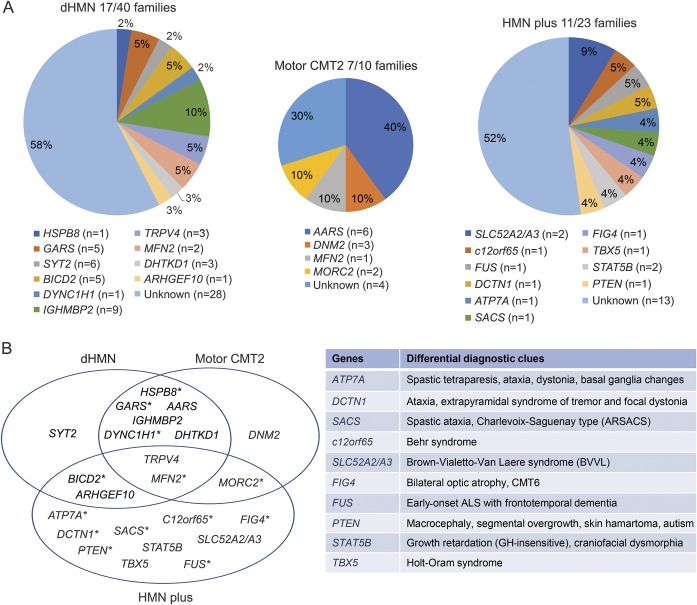
Identified genes in our hereditary motor neuropathy (HMN) patient cohort (A) Spectrum and distribution of mutated genes detected in our cohort within the 3 phenotypic groups. (B) Overlapping clinical phenotypes related to the identified genes and key additional clinical features associated. *Upper motor neuron involvement. ALS = amyotrophic lateral sclerosis.

**Figure 3:**
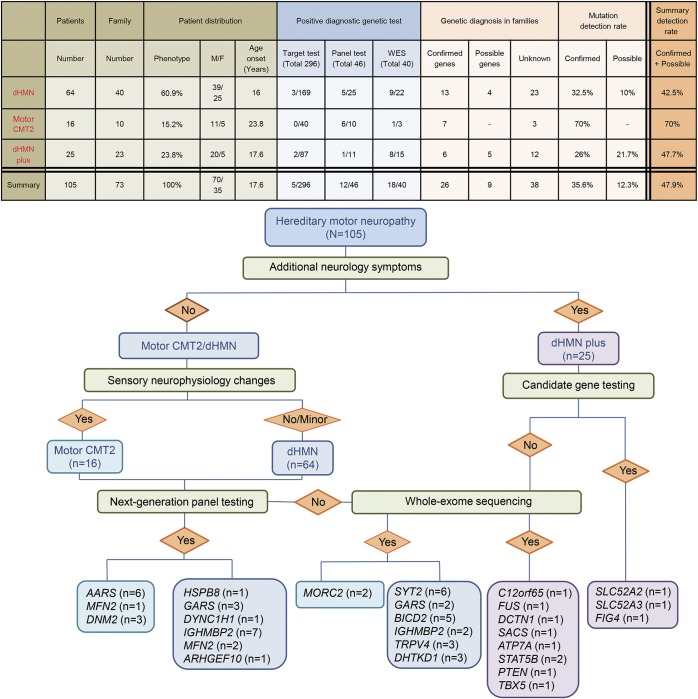
Diagnostic flow chart and mutation detection rates in our hereditary motor neuropathy (HMN) cohort CMT = Charcot-Marie-Tooth disease; dHMN = distal hereditary motor neuropathy; WES = whole exome sequencing.

Sixty-four out of 105 patients (60.9%) had dHMN. Sixteen patients had motor CMT2 (15.2%) and 25 HMN plus (23.8%) (figures 1–3). There was male predominance in all phenotypic groups. Symptoms started at a younger age in dHMN (mean 16 years) and HMN plus (mean 17.6 years) compared to motor CMT2 (mean 23.8 years). The main inheritance pattern was autosomal dominant in CMT2 (7/10 families [70%]) while isolated cases were more frequent in HMN plus. The dHMN group consisted of an almost equal number of dominant families (n = 16) and isolated patients with negative or unavailable family history (n = 20).

We identified causative mutations in 26 out of 73 families, providing a 35.6% mutation detection rate (figure 2). Candidate gene sequencing based on clinical presentation led to the molecular diagnosis in 5 out of 105 patients only (4.7%), while 12 index patients were diagnosed by NGS panel from 46 performed tests (26%) and a further 18 index patients by WES (total 40 analysis) (45%). There were no novel shared genes/mutations in the exome negative cases.

### Mutation spectrum in dHMN.

We achieved the possible genetic diagnosis in 17/40 families, which resulted in 42.5% detection rate (figure 2). Variants were considered confirmed pathogenic in 13 index cases (32.5%); 7 of them carried known pathogenic dHMN mutations while in 6 families novel deleterious variants were detected in known genes and segregated with the phenotype (table e-1A at Neurology.org).

A previously reported heterozygous c.421A>G, p.(Lys141Glu) mutation in *HSPB8* presented with a progressive lower limb predominant phenotype (family 1). We found 2 novel *GARS* mutations in 2 independent families (families 2–3). The heterozygous c.647A>G, p.(His216Arg) was detected in a mother and her son with predominant upper extremity involvement (dHMN-V) (figure 1A). The c.1528A>C, p.(Lys510Gln) mutation cosegregated in 3 generations with dominant distal motor neuropathy affecting both upper and lower extremities. Electrophysiologic examination revealed mildly increased jitter in one patient consistent with a motor neuropathy and significantly increased jitter with blocking in another patient. One patient with dHMN involving both upper and lower limbs carried the previously reported c.1834G>A, p.(Val612Met) mutation in *DYNC1H1* (family 7) (figure 1F). A rapidly progressive dHMN in a 15-year-old boy was due to the de novo previously reported c.1126A>G, p.(Met376Val) mutation in *MFN2* (family 12).^11^ The heterozygous c.2119C>T p.(Arg707Trp) *MFN2* mutation was found in a 70- year-old man with late-onset dHMN (family 13).^12^

The homozygous frameshift c.292_303delinsATGCT p.(Gly98fs) mutation in *IGHMBP2* led to SMARD1 in 2 siblings of consanguineous Pakistani origin (family 8) (figure 1B). A brother and sister from another family with childhood-onset distal SMA and lack of respiratory involvement carried a heterozygous c.1813C>T p.(Arg605*) nonsense *IGHMBP2* mutation, which was hemizygous in the cDNA (family 9).^13^

We have previously reported 6 patients with nonprogressive neuropathy and fatigable weakness carrying c.923C>T; p.(Pro308Leu) in *SYT2* (family 4) (figure 1G),^14^ 5 patients with distal congenital nonprogessive SMA carrying c.320C>T, p.(Ser107Leu) in *BICD2* (families 5–6) (figure 1H),^15^ and 2 patients with *TRPV4* mutations associated with childhood-onset scapuloperoneal SMA and metatropic dysplasia (family 10) (figure 1C) or adult-onset dHMN (family 11).^16^

Possibly causative mutations were detected in 4 families (table e-1B). The heterozygous novel c.2752C > T, p.(Arg918Cys) *IGHMBP2* variant was detected in 3 siblings with motor neuropathy, without respiratory dysfunction (family 14). All prediction tools predicted this change pathogenic, and it was absent in the ExAC database, although we could not identify a second mutation in this family. The single heterozygous c.767C>G, p.(Ala256Gly) *IGHMBP2* variant was detected in a 3.5-year-old boy with compatible phenotype (family 15) (figure 1D). In 3 members of a dominant dHMN pedigree (family 16), we found a novel heterozygous *DHTKD1* variant c.628G>T, p.(Ala210Ser). In a young female patient (family 17) we identified a heterozygous novel c.1949G>A, p.(Tyr650Cys) sequence change in *ARHGEF10*.

### Mutation spectrum in motor CMT2.

Motor CMT2 was considered in 16 patients from 10 families. We achieved 70% mutation detection rate in this group by concluding molecular diagnosis in 7 CMT2 families (figure 2) (table e-2). We identified the recurrent c.986G>A, p.(Arg329His) *AARS* mutation in 6 patients from 4 families (families 18–21) (figure 1, K and L).^17^ In 3 members of a dominant family with early-onset intermediate motor neuropathy, split hand deformity, and hearing loss, we detected the previously described c.1739T>C, p.(Met580Thr) *DNM2* mutation (family 22) (figure 1M).^18^ A female patient with upper limb weakness carried the pathogenic c.1403G>A, p.(Arg468His) *MFN2* mutation (family 23).^12^ Exome sequencing of male monozygotic twins with early-onset severe motor predominant neuropathy revealed the recently reported heterozygous c.754C>T, p.(Arg252Trp) mutation in the *MORC2* gene (family 24).^19^

### Mutation spectrum in HMN plus families.

**Figure 4:**
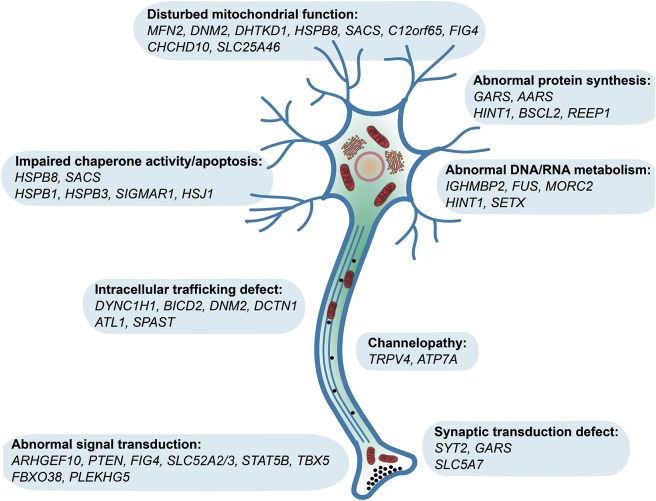
Pathomechanisms of genes reported in hereditary motor neuropathy (HMN)

Twenty-five patients from 23 families with predominant motor neuropathy had additional neurologic features. Confirmed pathogenic mutations were detected in 6 patients (26%) and a probable disease cause was identified in a further 5 index patients (21.7%) (figure 2, table e-3A). The heterozygous c.1529A>G p.(Lys510Arg) *FUS* mutation was detected in a 52-year-old man with asymmetric lower limb motor neuropathy. The progression was rapid with evolving motor neuron disease, frontal dementia, and loss of ambulation within half a year (family 27) (figure 1N). WES identified a hemizygous c.2279A>G, p.(Tyr760Cys) novel *ATP7A* variant with upper and lower motor neuron symptoms (family 29) (figure 1O).^20^ We detected compound heterozygous c.916G>A, p.(Gly306Arg) and c.1016T>C, p.(Leu339Pro) mutations in *SLC52A2* (family 25)^21^ and a homozygous c.96_99dupATCC, p.(Pro34Ilefs*25) mutation in the *c12orf65* gene (family 26).^22^ Distal motor neuropathy with ataxia and dystonia was associated with c.3823C>T p.(Arg1275Cys) in *DCTN1* (family 28).^23^ Compound heterozygous c.1580C>G, p.(Ser527*) and c.6781C>A, p.(Leu2261Ile) mutations in *SACS* caused juvenile-onset motor neuropathy, ataxia, and spasticity in a 71-year-old man (family 30).^24^ Possibly causative mutations were detected in 5 families (table e-3B). A 17-year-old man with distal motor neuropathy and optic atrophy (CMT type 6) carried a single previously reported heterozygous c.2386C>T, p.(Gln796*) mutation in *FIG4*.^25^ Heterozygous *FIG4* mutations have been associated with adult-onset amyotrophic lateral sclerosis but without optic atrophy.^26^ We found the heterozygous c.1371C>G, p.(Phe457Leu) mutation in *SLC52A3* in a 19-year-old man with Brown-Vialetto-Van-Laere syndrome, who responded well to riboflavin therapy. However, we could not identify a second mutation, even by analyzing WES for copy number variations within this gene (family 32). A 19-year-old man with shoulder girdle weakness commonly seen in Holt-Oram syndrome carried the previously reported c.331G>T, p.(Asp111Tyr) *TBX5* mutation (family 33, figure 1P).^27^ We identified the novel homozygous c.944T>G, p.(Glu315Ala) mutation in the transcriptional factor *STAT5B* in a sibling pair from a consanguineous family with growth retardation, dysmorphic face, and motor neuropathy (family 34) (figure 1Q). Asymmetric motor neuron weakness, cranial nerve involvement, pyramidal signs, and multifocal motor neuropathy with conduction blocks were observed in a man who carried a novel de novo c.269T>C, p.(Phe90Ser) mutation in *PTEN* (family 35).

## DISCUSSION

Although HMNs are suggested to be rare, we identified 105 patients in the North of England, with a population of 2.99 million. We previously determined that the minimum prevalence of CMT in the North of England is 11.8 per 100,000 individuals.^28,29^ Here we report that the minimum prevalence of dHMN in the same population is 2.14 affected individuals per 100,000 inhabitants (95% CI 1.62–2.66), which is higher than suggested earlier.^29,30^

Previous studies indicated that the molecular cause can be identified in the vast majority of patients with demyelinating CMT; however, the detection rate has been much lower in dHMN and motor CMT2 (∼20).^3^ We identified potentially pathogenic mutations in 47.9% (35/73 families), which consisted of confirmed mutations in 35.6% and possibly causative variants in an additional 12.3%. Previously in dHMN, the detection rate was 15%–20%.^3,5,6,31^ We confirmed genetic diagnosis in 32.5% (13/40 families) of dHMN, and a likely causative mutation was identified in an additional 10% (figure 2), which is higher than in previous studies (tables e-4–e-6).

dHMN is strictly considered as the pure motor end of CMT, many patients show minor sensory abnormalities,^1,6^ and mutations in the same gene can cause both dHMN and CMT2.^2,31^ HMN may be complicated by SMA, spastic paraplegia, or other neurologic abnormalities.^1,6,32^ Affected pathways linked to dHMN include DNA/RNA metabolism, protein translation and synthesis, stress response, apoptosis, axonal guidance, intracellular trafficking, and synaptic activity (figure 4). Mitochondrial abnormalities are common in our cohort in all forms of motor neuropathies and contribute to the symptoms by altering mitochondrial fusion/fission (*MFN2, DNM2, SLC25A46*), axonal transport (*HSPB1, HSPB8*), protein synthesis (*C12orf65, SACS*), or cofactors (*SLC52A2/3*).

SMA-LED has been introduced to characterize the congenital or early childhood-onset nonprogressive atrophy and weakness of the legs affecting both distal and proximal muscles, caused by altered motor neuron development or function.^3^ The clinical presentation may overlap between spinal and distal motor neuron dysfunction. Correction of deformities is important, since these patients do not deteriorate in the later disease course. We identified 17 patients with clinical symptoms resembling SMA-LED, carrying mutations in *SYT2*, *BICD2*, and *DYNC1H1*, and no causative mutation was identified in 4 patients (figure 1, I–J). No major common pathway can be highlighted by the pathomechanism of these genes, while different mutations in the same gene (such as *DYNC1H1*) may also cause different clinical presentations.

We reported that mutations in synaptic proteins can cause nonprogressive motor neuropathy and fatigable weakness with presynaptic neuromuscular junction (NMJ) defect (*SYT2*),^15^ which can benefit from 3,4 diaminopyridine treatment.^33^ Abnormal neuromuscular transmission has been shown in *Drosophila* and mouse models of *GARS* mutations.^34,35^ Furthermore, a genetic defect of the presynaptic choline transport leads to HMN due to *SLC5A7* mutations.^36^ Based on these findings, we studied neuromuscular transmission in 27 patients and substantial abnormalities (over and above that expected in the context of immature NMJs formed during denervation and reinnervation) were detected in 7 cases harboring different gene mutations. Further detailed studies of the NMJ are warranted, including genetically characterized patients. Neuromuscular transmission can be modified by several, already available compounds, which have been broadly used in congenital myasthenic syndromes, but not in hereditary neuropathies. We suggest performing repetitive stimulation and single fiber EMG as part of routine electrophysiologic studies in patients with HMN in order to uncover potentially treatable transmission defects.

We identified a subgroup of patients with inherited motor neuropathy, resembling multifocal demyelinating motor neuropathy or nodo-paranodopathy. Talipes and pure motor nerve involvement were caused by a novel mutation in *ARHGEF10*. Slow nerve conduction velocities have been implicated in *ARHGEF10* mutations and a 10-bp deletion in Leonberger dogs led to pelvic muscle weakness, stepping gait, and recurrent laryngeal nerve palsy.^37^

A previously reported pathogenic mutation in *TBX5* was detected in a patient with demyelinating multifocal motor neuropathy. *TBX5* participates in transcriptional regulatory cascades and its pathogenic mutations, located within the DNA binding T-box domain, manifest in a rare condition called Holt-Oram syndrome with deformity of upper limbs, carpal tunnel syndrome, and cardiac anomalies.^27^ We suggest that *TBX5* may cause demyelinating neuropathy affecting predominantly the motor nerves. No cardiac symptoms were detected in our patient. We detected a novel possibly causative *STAT5B* mutation in a sibling pair with motor neuropathy, ptosis, short stature, and dysmorphic facial features. Homozygous mutations in *STAT5B*, a signal transducer and activator of transcription, have been reported in only a handful of patients worldwide, all manifesting with postnatal growth retardation and occasional immunologic alterations.^38^ Impaired *STAT5B* signaling may lead to aberrant peripheral myelination through cyclin D1 overexpression and may have an effect on neuronal growth and differentiation.^39^ Finally, we detected a possibly causative de novo *PTEN* mutation in a patient with patchy motor neuropathy, focal demyelination, hamartoma-like skin lesions, and autism spectrum disorder. *PTEN*, a lipid phosphatase, inhibits phosphoinositide 3-kinase, which is also impaired in several forms of CMT (*FIG4, FGD4, MTMR2, MTMR13/SBF2*). It has been implicated in peripheral neural plasticity, axonal outgrowth, and hypermyelination. Experimental *PTEN* suppression in mice resulted in progressive peripheral neuropathy with tomacula formation and myelin outfoldings.^40^

The identification of pathogenic mutations in the same genes in dHMN and motor CMT indicate that dHMN should not be classified as a different disease group. The detection of mutations in rare disease genes, not implicated in neuropathy to date, suggest that the neuropathy can be part of a more complex genetic disease and key clinical signs are important to recognize these diseases. These overlapping phenotypes highlight basic biological pathways in peripheral nerves that may be targeted to develop therapies for patients with inherited motor neuropathies.

## Supplementary Material

Data Supplement

## GLOSSARY

CI: confidence interval
CMT: Charcot-Marie-Tooth disease
dHMN: distal hereditary motor neuropathy
HMN: hereditary motor neuropathy
LED: lower extremity dominance
MAF: minor allele frequency
NCS: nerve conduction studies
NGS: next-generation sequencing
NMJ: neuromuscular junction
SMA: spinal muscular atrophy
SMARD1: spinal muscular atrophy with respiratory distress
WES: whole exome sequencing
