# Supplementary material for: Genetic heterogeneity of motor neuropathies
Source: Neurology. 2017 Mar 28;88(13):1226–34. doi: 10.1212/WNL.0000000000003772 (PMC5373778; doi:10.1212/WNL.0000000000003772)
Supplement: Data Supplement [file supp_88_13_1226__index.html]

Genetic heterogeneity of motor neuropathies — Data Supplement 

# Genetic heterogeneity of motor neuropathies

## Data Supplement

**Neurology® data supplements are not copyedited before publication. Published editorials and translations have been copyedited.  
 © 2017 American Academy of Neurology.  
  
 Files in this Data Supplement:**

- e-Tables - PDF
