# Supplementary material for: Genetic heterogeneity of motor neuropathies
Source: Neurology. 2017 Mar 28;88(13):1226–34. doi: 10.1212/WNL.0000000000003772 (PMC5373778; doi:10.1212/WNL.0000000000003772)
Supplement: Data Supplement [file supp_WNL.0000000000003772_e-tables.pdf]

Suppl. Table e-1. Summary of the clinical presentation of the patients in the dHMN group **A)** Confirmed causative mutations

Family / Patient / Age/ Sex	***Gene***	***Nucleotide / Amino acid change***	***Phenotype***	***Inherit***	***Age onset***	***Lower limb***				***Upper limb***				***Deformity / Contracture***	***Walking / Orthotics***	***Additional features***
						**Weakness**	**Wasting**	**Reflexes ankle /knee**	**Sensory**	**Weakness**	**Wasting**	**Reflexes**	**Sensory**			
F1 / P1<br>26y / f	HSPB8	c.421A>G<br>p.(Lys141Glu)	dHMN-II	AD	10y	d ++	d +	a /↑	pain	d +	-	N	-	pes cavus	HW /<br>AFO	-
F2 / P2<br>36y / m	GARS	c.647A>G<br>p.(His216Arg)	dHMN-V<br>upper limb	AD	14y	d +	d +	↓/n	+	d +++	d+++	A	+	split hand<br>pes cavus	-	swollen tongue
F2 / P3<br>66y / f	GARS	c.647A>G<br>p.(His216Arg)	dHMN-V<br>upper limb	AD	12y	d +	d +	↓/↓	+	d +++	d +++	A	+	split hand	-	swollen tongue<br>immune arthritis
F3 / P4<br>51y / f	GARS	c.1528A>C<br>p.(Lys510Gln)	dHMN-V<br>lower limb	AD	5y	d ++	d ++	a /↑	pain	d ++	d ++	↑	pain	pes cavus /<br>tight Achilles	tiptoeing,<br>HW	hearing deficit
F3 / P5<br>9y / f	GARS	c.1528A>C<br>p.(Lys510Gln)	dHMN-V<br>lower limb	AD	5y	d +	d +	↓/ ↑	-	-	-	N	-	tight Achilles	tiptoeing,<br>falls	-
F3 / P6<br>28y / f	GARS	c.1528A>C<br>p.(Lys510Gln)	dHMN-V<br>lower limb	AD	6y	d ++	d ++	a / n	+	d +	d +	N	+	pes cavus /<br>tight Achilles	tiptoeing,<br>HW	-
F4/ P7<br>46y / f	SYT2	c.923C>T<br>p.(Pro308Leu)	SMA-LED	AD	birth	R d +++<br>p ++<br>L d +++	R d +++<br>p ++<br>L d +++	a / a	+	d +	-	A	+	pes cavus<br>splayed toes /<br>hip dysplasia	HW,TW /<br>knee splint	-
F4/ P8<br>29y / f	SYT2	c.923C>T<br>p.(Pro308Leu)	SMA-LED	AD	birth	d +++	d +++	a / a	+	d +	-	A	+	pes cavus<br>hammer toes /<br>hip dysplasia	waddling /<br>AFO	-
F4/ P9<br>8y / m	SYT2	c.923C>T<br>p.(Pro308Leu)	SMA-LED	AD	birth	-	-	a / a	+	-	-	A	-	pes planus	HW /<br>insoles	motor delay,<br>ADHD
F4/ P10<br>17y / m	SYT2	c.923C>T<br>p.(Pro308Leu)	SMA-LED	AD	birth	-	-	a / a	-	-	-	A	-	pes cavus<br>hammer toes	-	-
F4/ P11<br>43y / m	SYT2	c.923C>T<br>p.(Pro308Leu)	SMA-LED	AD	birth	d +++	d +++	a / a	-	-	-	A	-	hammer toes	HW, TW	reflex<br>facilitation<br>on exercise
F4/ P12<br>14y / f	SYT2	c.923C>T<br>p.(Pro308Leu)	SMA-LED	AD	birth	R d ++<br>L d +	-	a / a	-	-	-	A	-	pes cavus<br>splayed toes /<br>hip dysplasia	HW	-
F5/ P13<br>19y / m	BICD2	c.320C>T<br>p.(Ser107Leu)	SMA-LED	isolated<br>de novo	birth	d +++	d ++	a /↓	-	-	d +	↓	-	club feet<br>scapular<br>winging	steppage	motor delay
F6/ P14<br>42y / f	BICD2	c.320C>T<br>p.(Ser107Leu)	SMA-LED	AD	birth	d +<br>p +	d ++	↑/ ↑	-	-	-	N	-	crowded toes<br>scapular<br>winging	difficulties	motor delay
F6/ P15<br>7y / f	BICD2	c.320C>T<br>p.(Ser107Leu)	SMA-LED	AD	birth	d +<br>p +	d ++	↑/ ↑	-	-	-	↑	-	pes<br>equinovarus,<br>scapular<br>winging	difficulties	motor delay

Abbreviations: F, family; P, patient; y, year; m, male; f, female; † , deceased; AD, autosomal dominant; AR, autosomal recessive; d, distal; p, proximal; R, right; L, left; +, mild; ++, moderate; +++, severe; a, absent; n, normal; ↑, increased; ↓, decreased; -, none; HW, impaired heel walking; TW, impaired toe walking; AFO, ankle foot orthesis; SGA, small for gestational age, SMA-LED, spinal muscular atrophy with lower extremity dominance

Suppl. Table e-1. Summary of the clinical presentation of the patients in the dHMN group **A)** Confirmed causative mutations

Family / Patient / Age/ Sex	***Gene***	***Nucleotide / Amino acid change***	***Phenotype***	***Inherit***	***Age onset***	***Lower limb***				***Upper limb***				***Deformity / Contracture***	***Walking / Orthotics***	***Additional features***
						**Weakness**	**Wasting**	**Reflexes ankle /knee**	**Sensory**	**Weakness**	**Wasting**	**Reflexes**	**Sensory**			
F6 / P16 6y / m	BICD2	c.320C>T p.(Ser107Leu)	SMA-LED	AD	birth	d + p +	d ++	↑/ ↑	-	-	-	↑	-	scapular winging	difficulties	motor delay
F6 / P17 6y / m	BICD2	c.320C>T p.(Ser107Leu)	SMA-LED	AD	birth	d + p +	d ++	↑/ ↑	-	-	-	↑	-	scapular winging	difficulties	motor delay
F7 / P18 28y / m	DYNC1H1	c.1834G>A p.(Val612Met)	SMA-LED	AD	6y	d +++	d +++	a /↓	-	-	-	n	-	pes cavus, crawled toes	HW,TW / splints	pyramidal signs
F8 / P19 3y† / m	IGHMBP2	c.292_303 delinsATGCT p.(Gly98fs)	SMARD1	AR	birth	d +++ p ++	d ++	a / a	-	d +++ p ++	d ++	a	-	pes cavus / flexed fingers tight Achilles	not developed	preterm, SGA, respiratory failure
F8 / P20 6mo†/ m	IGHMBP2	c.292_303 delinsATGCT p.(Gly98fs)	SMARD1	AR	birth	d +++	d +	a / a	-	d +++	d +	a	-	pes cavus	not developed	preterm, SGA, respiratory failure
F9 / P21 21y / m	IGHMBP2	c.1813C>T p.(Arg605*)	dHMN	AR	7y	d +++ p +++	d +++ p ++	a /↓	-	d ++	d +	n	-	pes cavus	ambulant with crutch, wheelchair	-
F9 / P22 19y / f	IGHMBP2	c.1813C>T p.(Arg605*)	dHMN	AR	10y	d ++	d ++	a / a	-	d +	d +	a	-	pes cavus	HW,TW / AFO	-
F10 / P23 9y / m	TRPV4	c.805C>T p.(Arg269Cys)	congenital dHMN	de novo	birth	d +++ p ++	d ++	a / a	-	-	-	↓	-	pes cavus, scapular winging / tight Achilles	HW,TW / splints, wheelchair	intermittent horse voice
F11/ P24 49y / f	TRPV4	c.184G>A p.(Asp62Asn)	congenital dHMN	AD	41y	d +++	d ++	a / a	-	-	-	n	-	pes cavus, crawled toes	HW,TW / splints, crutches	livid skin
F11 / P25 43y / m	TRPV4	c.184G>A p.(Asp62Asn)	congenital dHMN	AD	birth	-	-	n	-	-	-	n	-	talipes	-	-
F12 / P26 16y / m	MFN2	c.1126 A>G p.(Met376Val)	dHMN	isolated de novo	14y	d +++	d ++	↑/↑	pain	-	-	↑	-	pes cavus, hammer toes / tight Achilles	HW tiptoeing	-
F13 / P27 70y / m	MFN2	c.2119C>T p.(Arg707Trp)	dHMN	isolated de novo	55y	R d +++ p +++	d ++	a / a	-	R d ++ p +++	R d +	a	-	-	non-ambulant / wheelchair	Alzheimer disease

Abbreviations: F, family; P, patient; y, year; m, male; f, female; AR, autosomal recessive; AD, autosomal dominant; d, distal; p, proximal; R, right; +, mild; ++, moderate; +++, severe; a, absent;↑ increased;↓, decreased; n, normal; -, none; HW, impaired heel walking; TW, impaired toe walking, SMA-LED, spinal muscular atrophy with lower extremity dominance; SMARD1, spinal muscular atrophy with respiratory distress

Suppl. Table e-1. Summary of the clinical presentation of the patients in the dHMN group **B)** Possibly causative mutations

Family / Patient / Age/ Sex	***Gene***	***Nucleotide / Amino acid change***	***Phenotype***	***Inherit***	***Age onset***	***Lower limb***				***Upper limb***				***Deformity / Contracture***	***Walking / Orthotics***	***Additional features***
						**Weakness**	**Wasting**	**Reflexes ankle /knee**	**Sensory**	**Weakness**	**Wasting**	**Reflexes**	**Sensory**			
F14 / P28 32y / f	IGHMBP2	c.2752 C>T p.(Arg918Cys)	dHMN	AR	6y	d ++	d ++	a / a	+	d ++	d ++	n	-	hammer toes	HW,TW / splints	-
F14 / P29 13y / m	IGHMBP2	c.2752 C>T p.(Arg918Cys)	dHMN	AR	4y	d ++	d ++	a / a	-	-	-	n	-	pes cavus	HW,TW / splints	-
F14 / P30 30y / m	IGHMBP2	c.2752 C>T p.(Arg918Cys)	dHMN	AR	10y	d ++	d ++	a / a	-	-	-	n	-	pes cavus	HW,TW / splints	-
F14 / P31 48y / m	IGHMBP2	c.2752 C>T p.(Arg918Cys)	dHMN	AR	7y	d +++	d +++	a / a	+++	d ++	d +	n	-	pes cavus	HW,TW / splints	-
F15 / P32 3,5y / m	IGHMBP2	c.767C>G p.(Ala256Gly)	dHMN	isolated	birth	d +++	d ++	a /↓	-	-	-	↓	-	pes cavus scapular winging	falls	motor delay
F16 / P33 72y / f	DHTKD1	c.628G>T p.(Ala210Ser)	dHMN	AD	10y	d ++	d +++	a / a	-	d ++	d ++	a	-	pes cavus, clawed toes	HW	'crane leg' ulnar split hand
F16 / P34 48y / m	DHTKD1	c.628G>T p.(Ala210Ser)	dHMN	AD	39y	d ++ p ++	d +++	a / a	+	d ++	d ++	a	+	pes cavus clawed toes	HW, TW / insoles	'crane leg' ulnar split hand
F16 / P35 45y / m	DHTKD1	c.628G>T p.(Ala210Ser)	dHMN	AD	21y	d ++	d +++	a / a	-	d +	d ++	a	-	pes cavus clawed toes	HW	'crane leg' ulnar split hand
F17 / P36 33y / f	ARHGEF10	c.1949G>A p.(Cys650Tyr)	dHMN	isolated	birth	d ++	d +	a / a	-	d ++	d +	a	-	arthrogryposis talipes / hip dyslocation	HW,TW	keratotis swallowing difficulties

Abbreviations: F, family; P, patient; y, year; m, male; f, female; AD, autosomal dominant; AR, autosomal recessive; R, right; L, left; d, distal ; p, proximal; +, mild; ++, moderate; +++, severe; a, absent; n, normal; ↑, increased; ↓, decreased; -, none; HW, impaired heel walking; TW, impaired toe walking; AFO, ankle foot orthesis; ADHD, attention deficit hyperactivity disorder; SMA-LED, spinal muscular atrophy with lower extremity dominance

Suppl. Table e-2. Summary of the clinical presentation of the patients and confirmed mutations in the motor CMT2 group

Family / Patient / Age/ Sex	*Gene*	*Nucleotide / Amino acid change*	*Inherit*	*Age onset*	*Lower limb*				*Upper limb*				*Deformity / Contracture*	*Walking/ Orthotics*	*Additional features*
					Weakness	Wasting	Reflexes ankle /knee	Sensory	Weakness	Wasting	Reflexes	Sensory			
F18 / P37 56y / m	AARS	c.986G>A, p.(Arg329His)	AD	50y	d +++	d ++	a / a	+	-	-	n	-	pes cavus / tight Achilles	HW,TW / AFO	Dupuytren's contracture
F18 / P38 32y / m	AARS	c.986G>A, p.(Arg329His)	AD	28y	d ++	d ++	a / a	++	d ++	d ++	a	++	pes cavus	HW,TW / AFO	JRA , bilateral hip replacement
F19 / P39 77y †/ m	AARS	c.986G>A, p.(Arg329His)	AD	60y	d +++	d +++	a / a	++	d ++	d ++	a	++	pes cavus toe drop	HW, TW / AFO crutches	-
F19 / P40 50y / m	AARS	c.986G>A, p.(Arg329His)	AD	30y	d ++	d ++	a / a	++	d ++	d ++	a	++	pes cavus split hand	stepping / stick hand splint	TIA
F20 / P41 20y / m	AARS	c.986G>A, p.(Arg329His)	AD	12y	d +++	d ++	a / a	+	-	-	n	-	pes cavo-equinus / tight Achilles	stepping / insoles	-
F21 / P42 54y / f	AARS	c.986G>A, p.(Arg329His)	AD	20y	d +++	d +++	a / a	++	d ++	d +	↓	-	pes cavus	HW,TW stepping / AFO	hearing deficit
F22 / P43 59y / m	DNM2	c.1739T>C p.(Met580Thr)	AD	5y	d +++	d +++	a / a	+	d +++	d +++	a	+	clawed toes curled fingers split hand	HW,TW / AFO	hearing deficit respiratory muscle weakness
F22 / P44 36y / f	DNM2	c.1739T>C p.(Met580Thr)	AD	11y	d +++	d +++	a / a	+	d +++	d +++	a	+	clawed toes curled fingers	HW,TW / AFO	-
F22 / P45 30y / f	DNM2	c.1739T>C p.(Met580Thr)	AD	13y	d ++	d ++	a / a	+	d ++	d ++	a	+	clawed toes	HW, TW / AFO	-
F23 / P46 35y / f	MFN2	c.1403G>A, p.(Arg468His)	AD	20y	d+	d+	a/a	+	d+++	d+++	a	+	clawed fingers	hand splint	-
F24 / P47 59 / m	MORC2	c.C754T, p.(Arg252Trp)	AD	13y	d+++	d+++	a/a	+++	d+++	d+++	a	++	pes cavus arthrodesis	non-ambulant	mild deafness
F24 / P48 59 / m	MORC2	c.C754T, p.(Arg252Trp)	AD	10y	d+++	d+++	a/a	+++	d+++	d+++	a	++	pes cavus arthrodesis	non-ambulant	mild deafness

Abbreviations: F, family; P, patient; y, year; m, male; f, female; AD, autosomal dominant; d, distal ; +, mild; ++, moderate; +++, severe; a, absent; n, normal; ↓, decreased; -, none; HW, impaired heel walking; TW, impaired toe walking; AFO, ankle foot orthesis; JRA, juvenile rheumatoid arthritis; TIA, transient ischaemic attack

Suppl. Table e-3. Summary of the clinical presentation of the patients in the HMN plus group **A)** Confirmed pathogenic mutations

Family / Patient / Age/ Sex	*Gene*	*Nucleotide / Amino acid change*	*Inherit*	*Age onset*	*Lower limb*				*Upper limb*				*Deformity / Contracture*	*Walking / Orthotics*	*Additional features*
					**Weakness**	**Wasting**	**Reflexes ankle /knee**	**Sensory**	**Weakness**	**Wasting**	**Reflexes**	**Sensory**			
F25 / P49<br>14y / f	SLC52A2	c.916G>A,<br>p.(Gly306Arg)<br>c.1016T>C,<br>p.(Leu339Pro)	AR	18mo	d +	d +	a / a	-	d +++<br>p +++	d +++	a	-	curled fingers	ataxic	optic atrophy<br>sensorineural hearing loss<br>bulbar palsy<br>axial weakness<br>ataxia
F26 / P50<br>25y / m	C12orf65	c.96_99dup<br>ATCC,<br>p.(Pro34Ilefs*25)	AR	2y	d +++	d ++	↑ / ↑	-	d ++	d ++	↑	-	pes cavus / tight Achilles	tiptoeing, HW, TW	optic atrophy<br>spasticity<br>pyramidal signs<br>autistic
F27 / P51<br>52y / m	FUS	c.1529A>G<br>p.(Lys510Arg)	AD	48y	d +++<br>p +++	d +++<br>p +++	↑ / ↑	-	-	-	↑	-	-	HW, TW / wheelchair	mild frontal dementia
F28 / P52<br>74y / m	DCTN1	c.3406C>T<br>p.(Arg1136Cys)	AD<br>de novo	63y	d +++<br>p ++	d +++	a /a	-	d +++<br>p +++	d +++<br>p +++	↑	-	pes cavus	HW, TW / AFO	cranial nerves<br>ataxia<br>dystonia
F29 / P53<br>29y / m	ATP7A	c.A2279G:<br>p.(Tyr760Cys)	X	10y	d ++	d ++	↑ / ↓	-	-	-	↑	-	-	HW, TW<br>spastico – ataxic / wheelchair	cranial nerves<br>tetraspasticity<br>pyramidal signs<br>extrapyramidal<br>ataxia
F30 / P54<br>71y / m	SACS	c.1580C>G,<br>p.(Ser527*)<br>c.6781C>A,<br>p.(Leu2261Ile)	AR	19y	d +++	d +++	a / ↑	+	d ++	d+++	↑	+	pes cavus<br>curled fingers	HW, TW<br>spastic - paraparetic	dysarthria<br>spasticity<br>ataxia<br>learning difficulties

Abbreviations: F, family; P, patient; y, year; mo, month; m, male; f, female; AD, autosomal dominant; AR, autosomal recessive; X, X-linked; R, right; L, left; d, distal ; p, proximal; +, mild; ++, moderate; +++, severe; a, absent;↑, increased; ↓, decreased; -, none; HW, impaired heel walking; TW, impaired toe walking; AFO, ankle foot orthesis

Suppl. Table e-3. Summary of the clinical presentation of the patients in the HMN plus group **B)** Possibly causative mutations

Family / Patient / Age/ Sex	*Gene*	*Nucleotide / Amino acid change*	*Inherit*	*Age onset*	*Lower limb*				*Upper limb*				*Deformity / Contracture*	*Walking / Orthotics*	*Additional features*
					**Weakness**	**Wasting**	**Reflexes ankle /knee**	**Sensory**	**Weakness**	**Wasting**	**Reflexes**	**Sensory**			

F31 / P55<br>25y / m	FIG4	c.2386C>T<br>p.(Gln796*)	AD	17y	d ++	d +++	a / ↑	++	-	-	↑	++	pes cavus<br>clawed toes	HW, TW	optic atrophy<br>pyramidal signs
F32 / P56<br>24y / m	SLC52A3<br><br>SLC52A2	c.1371C>G,<br>p.(Phe457Leu)<br>c.819C>T<br>p.(Met273Ile)	AR	19y	d ++	d ++	a / a	+	d +++<br>p +++	d +++<br>p +	a	+	curled<br>fingers	ataxic<br>HW, TW /<br>wheelchair	ophthalmoplegia<br>sensorineural<br>hearing loss<br>bulbar palsy<br>ataxia<br>respiratory failure
F33 / P57<br>19y / m	TBX5	c.331G>T,<br>p.(Asp111Tyr)	AD	2y	d ++	d ++	↑ / ↑	+	d ++	d +	↑	+	pes cavus<br>clawed toes	clumsy	shoulder deformity<br>thoracic kyphosis<br>scapular winging
F34 / P58<br>16y / m	STAT5B	c.944A>C,<br>p.(Glu315Ala)	AR	10y	d ++	d ++	↓ / ↓	-	-	-	↓	-	-	HW, TW	postnatal growth<br>retardation<br>face dysmorphia<br>ptosis<br>facial weakness
F34 / P59<br>17y / f	STAT5B	c.944A>C,<br>p.(Glu315Ala)	AR	10y	d ++	d +	↓ / ↓	-	-	-	↓	-	-	HW, TW	postnatal growth<br>retardation<br>face dysmorphia<br>ptosis<br>facial weakness
F35 / P60<br>29y / m	PTEN	c.269T>C<br>p.(Phe90Ser)	isolated	3y	R d +++	R d ++	↑ / ↑	-	L d +++<br>p +++<br>R d ++	L d +++<br>p +++<br>R p ++	a	-	pes cavus<br>clawed toes	falls,<br>HW,TW	cranial nerve III.,<br>IV., VII., X.,XII.<br>macrocephaly<br>development delay

Abbreviations: F, family; P, patient; y, year; m, male; f, female; AD, autosomal dominant; AR, autosomal recessive; X, X-linked; R, right; L, left; d, distal ; p, proximal; +, mild; ++, moderate; +++, severe; a, absent;↑, increased; ↓, decreased; -, none; HW, impaired heel walking; TW, impaired toe walking; AFO, ankle foot orthesis

Suppl. Table e-4. Coverage of rare genetic variants from WES

Sample ID(s)	Average Read Depth(s)	Gene Name	Ensembl Gene ID	Ensembl Transcript ID	Exon	chromosome	genomic (hg19)	cDNA	protein	Genotype
F2/P2	51.2	GARS	ENSG00000106105	ENST00000389266	5	7	g.30642727A>G	c.A647G	p.H216R	Heterozygous
F3/P4-5	55.4, 57.5	GARS	ENSG00000106105	ENST00000389266	12	7	g.30661993A>C	c.A1528C	p.K510Q	Heterozygous
F4/P8-P9	77.1, 75.9	SYT2	ENSG00000143858	ENST00000367268	8	1	g.202568476G>A	c.C923T	p.P308L	Heterozygous
F5/P13,F6/P14-15	76.9, 70.5, 68.2	BICD2	ENSG00000185963	ENST00000375512	2	9	g.95491439G>A	c.C320T	p.S107L	Heterozygous
F9/P21-22	78.6, 72.8	IGHMBP2	ENSG00000132740	ENST00000255078	13	11	g.68703761C>T	c.C1813T	p.R605X	Heterozygous
F10/P23	73.3	TRPV4	ENSG00000111199	ENST00000538125	5	12	g.110238471G>A	c.C805T	p.R269C	Heterozygous
F11/P24-25	43.4	TRPV4	ENSG00000111199	ENST00000538125	2	12	g.110252418C>T	c.G184A	p.D62N	Heterozygous
F16/P34-35-36	80.9, 82.0, 48.1	DHTKD1	ENSG00000181192	ENST00000263035	4	10	g.12129639G>T	c.G628T	p.A210S	Heterozygous
F24/ P47	85.1	MORC2	ENSG00000133422	ENST00000215862	10	22	g.31337490G>A	c.C568T	p.R190W	Heterozygous
F26/P50	114.8	C12orf65	ENSG00000130921	ENST00000546132	3	12	g.123738316_123738317insATCC	c.95_96insATCC	p.L32fs	Homozygous
F27/P51	63.5	FUS	ENSG00000089280	ENST00000568685	14	16	g.31202419A>G	c.A1532G	p.K511R	Heterozygous
F28/P52	74.7	DCTN1	ENSG00000204843	ENST00000361874	32	2	g.74588640G>A	c.C3823T	p.R1275C	Heterozygous
F29/P53	72.1	ATP7A	ENSG00000165240	ENST00000341514	10	X	g.77268482A>G	c.A2279G	p.Y760C	Homozygous
F30/P54	75.1	SACS	ENSG00000151835	ENST00000382298	10	13	g.23911234G>T	c.C6781A	p.L2261I	Heterozygous
F30/P54	75.1	SACS	ENSG00000151835	ENST00000382298	8	13	g.23929171G>C	c.C1580G	p.S527X	Heterozygous
F31/P55	55.0	FIG4	ENSG00000112367	ENST00000230124	21	6	g.110113794C>T	c.C2386T	p.Q796X	Heterozygous
F32/P56	51.8	SLC52A3	ENSG00000101276	ENST00000217254	5	20	g.741709G>C	c.C1371G	p.F457L	Heterozygous
F33/P57	46.2	TBX5	ENSG00000089225	ENST00000405440	4	12	g.114837349C>A	c.G331T	p.D111Y	Heterozygous
F34/P58-59	70.3, 84.0	STAT5B	ENSG00000173757	ENST00000293328	8	17	g.40370786T>G	c.A944C	p.E315A	Homozygous
F35/P60	67.1	PTEN	ENSG00000171862	ENST00000371953	5	10	g.89692785T>C	c.T269C	p.F90S	Heterozygous

Suppl. Table e-5. Population frequencies of rare genetic variants from WES

SAMPLE INFO	DETAILS OF VARIATION		POPULATION ALLELE FREQUENCIES / IDS				
**Sample ID(s)**	**Gene Name**	**protein**	**ExAC**	**NHLBI_ESP6500**	**cg69**	**InHouse_281**	**dbSNP142**
F2/P2	GARS	p.H216R	8.28E-06	.	.	0.00178	.
F3/P4-5	GARS	p.K510Q	.	.	.	0.00356	.
F4/P8-P9	SYT2	p.P308L	.	.	.	0.00356	.
F5/P13,F6/P14-15	BICD2	p.S107L	.	.	.	0.00534	rs398123028
F9/P21-22	IGHMBP2	p.R605X	.	.	.	0.00356	.
F10/P23	TRPV4	p.R269C	.	.	.	0.00178	rs267607146
F11/P24-25	TRPV4	p.D62N	8.44E-06	.	.	0.00178	.
F16/P34-35-36	DHTKD1	p.A210S	0.0029	0.0028	.	0.00890	rs146741810
F24/ P47	MORC2	p.R190W	.	.	.	0.00178	.
F26/P50	C12orf65	p.L32fs	4.13E-05	.	.	0.01068	rs765675424
F27/P51	FUS	p.K511R	.	.	.	0.00178	.
F28/P52	DCTN1	p.R1275C	8.29E-06	.	.	0.00178	.
F29/P53	ATP7A	p.Y760C	4.56E-05	.	.	0.00356	.
F30/P54	SACS	p.L2261I	0.0048	0.0037	.	0.00356	rs146722795
F30/P54	SACS	p.S527X	.	.	.	0.00178	.
F31/P55	FIG4	p.Q796X	.	.	.	0.00178	.
F32/P56	SLC52A3	p.F457L	0.0005	0.0005	.	0.00178	rs145431028
F33/P57	TBX5	p.D111Y	0.0034	0.0034	.	0.00534	rs77357563
F34/P58-59	STAT5B	p.E315A	0.0027	.	.	0.00712	rs572536541
F35/P60	PTEN	p.F90S	.	.	.	0.00178	.

Suppl. Table e-6. Functional prediction of rare genetic variants from WES

SAMPLE INFO	DETAILS OF VARIATION		FUNCTIONAL PREDICTIONS / SCORES								
Sample ID(s)	Gene Name	protein	SIFT	Polyphen2	LRT	MutationTaster	MutationAssessor	FATHMM	VEST3 score	CADD (phred)	SiPhy 29way (log Odds)
F2/P2	GARS	p.H216R	D	D	D	D	M	T	0.908	22.4	10.631
F3/P4-5	GARS	p.K510Q	T	B	D	D	L	D	0.507	18.38	13.659
F4/P8-P9	SYT2	p.P308L	D	D	N	D	H	T	0.953	25.6	17.948
F5/P13,F6/P14-15	BICD2	p.S107L	T	D	D	D	M	T	0.787	28.7	16.783
F9/P21-22	IGHMBP2	p.R605X	T	.	D	D	.	.	.	25	12.81
F10/P23	TRPV4	p.R269C	T	D	D	D	N	D	0.957	13.58	12.232
F11/P24-25	TRPV4	p.D62N	D	D	U	D	L	D	0.201	18.86	14.004
F16/P34-35-36	DHTKD1	p.A210S	D	B	D	D	N	T	0.287	11.18	14.921
F24/ P47	MORC2	p.R190W	D	D	D	D	L	T	0.936	19.12	14.304
F26/P50	C12orf65	p.L32fs	.	.	.	.	.	.	.	.	.
F27/P51	FUS	p.K511R	D	D	D	D	M	D	0.844	15.36	10.436
F28/P52	DCTN1	p.R1275C	D	D	D	D	N	T	0.654	31	18.468
F29/P53	ATP7A	p.Y760C	D	D	D	D	H	D	0.97	21.4	14.894
F30/P54	SACS	p.L2261I	D	P	D	D	M	D	0.688	18.64	19.949
F30/P54	SACS	p.S527X	T	.	D	A	.	.	.	40	20.024
F31/P55	FIG4	p.Q796X	T	.	D	A	.	.	.	40	18.787
F32/P56	SLC52A3	p.F457L	D	D	D	D	M	T	0.978	17.17	10.944
F33/P57	TBX5	p.D111Y	D	D	D	D	M	D	0.936	22.9	18.282
F34/P58-59	STAT5B	p.E315A	T	B	D	D	L	T	0.354	13.9	9.432
F35/P60	PTEN	p.F90S	.	D	D	D	H	D	0.968	23.8	14.841
